# Time‐restricted feeding mediated modulation of microbiota leads to changes in muscle physiology in Drosophila obesity models

**DOI:** 10.1111/acel.14382

**Published:** 2024-10-24

**Authors:** Christopher Livelo, Yiming Guo, Jagathnarayan Madhanagopal, Casey Morrow, Girish C. Melkani

**Affiliations:** ^1^ Division of Molecular and Cellular Pathology, Department of Pathology, Heersink School of Medicine University of Alabama at Birmingham Birmingham Alabama USA; ^2^ Department of Cell, Developmental, and Integrative Biology University of Alabama at Birmingham Birmingham Alabama USA; ^3^ UAB Nathan Shock Center 1300 University Boulevard Birmingham Birmingham Alabama USA

**Keywords:** 16S microbiome analysis, ATP, Drosophila obesity models, insulin sensitivity and resistance, metabolic homeostasis, muscle metabolism, skeletal muscle physiology, time‐restricted feeding

## Abstract

Recent research has highlighted the essential role of the microbiome in maintaining skeletal muscle physiology. The microbiota influences muscle health by regulating lipid metabolism, protein synthesis, and insulin sensitivity. However, metabolic disturbances such as obesity can lead to dysbiosis, impairing muscle function. Time‐restricted feeding (TRF) has been shown to mitigate obesity‐related muscle dysfunction, but its effects on restoring healthy microbiomes remain poorly understood. This study utilizes 16S microbiome analysis and bacterial supplementation to investigate the bacterial communities influenced by TRF that may benefit skeletal muscle physiology. In wild‐type and obese *Drosophila* models (axenic models devoid of natural microbial communities), the absence of microbiota influence muscle performance and metabolism differently. Specifically, axenic wild‐type *Drosophila* exhibited reduced muscle performance, higher glucose levels, insulin resistance, ectopic lipid accumulation, and decreased ATP levels. Interestingly, in obese *Drosophila* (induced by a high‐fat diet or predisposed obesity mutant Sk2), the absence of microbiota improved muscle performance, lowered glucose levels, reduced insulin resistance, and increased ATP levels. TRF was found to modulate microbiota composition, notably increasing *Acetobacter pasteurianus* (AP) and decreasing *Staphylococcus aureus* (SA) in both obesity models. Supplementation with AP improved muscle performance and reduced glucose and insulin resistance, while SA supplementation had the opposite effect. This study provides novel insights into the complex interactions between TRF, microbiota, and skeletal muscle physiology in different *Drosophila* models.

## Graphical abstract

Time‐restricted feeding (TRF) impacts skeletal muscle physiology and metabolism through microbiome modulation. In Drosophila models, TRF altered microbiota composition, increasing beneficial *Acetobacter pasteurianus* (AP) and reducing harmful Staphylococcus aureus (SA). AP supplementation improved muscle performance and metabolic health, while SA worsened them. This study highlights the complex interactions between TRF, microbiota, and muscle health, offering insights into obesity‐related muscle dysfunction and potential therapeutic strategies.

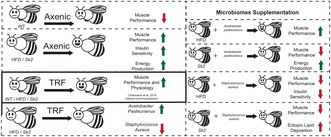

**Abbreviations**
AA: axenic antibiotic
Acc2: Acetyl‐CoA carboxylase 2
ALF: Ad libitum feeding
AMPK: AMP‐activated protein kinase
AP: *Acetobacter pasteurianus*
AS: axenic sterile
ASV: amplicon sequence variant
ATCC: American Type Culture Collection
Con: control
CS: canton‐S
DEG: differentially expressed genes
FoxO1: forkhead box protein O1
Glut1: glucose‐transporter 1
HFD: high‐fat diet
IFMs: indirect flight muscles
IMCL/IMTG: intramyocellular lipids/intramyocellular triglycerides
LD: lipid droplet
Myod1: myogenic differentiation 1
NLaz: neural lazarillo
Ppargc1: PPARG coactivator 1 alpha
QIIME2: quantitative insights for microbial ecology 2
RNA‐seq: RNA‐sequencing
RPL11: ribosomal protein L11
SA: *Staphylococcus aureus*
Sk2: sphingosine kinase 2
TRF: time‐restricted feeding

## 1 INTRODUCTION

The microbiome comprises living members including bacteria, archaea, fungi, algae, and protists (Berg et al., 2020). The microbiome is further defined not only by its community of microorganisms but inclusive by its interactions involving molecular products of microorganisms, metabolites, and surrounding environmental conditions (Berg et al., 2020). In many living organisms including humans, the microbiota can play a crucial role in regulating metabolism, immunity, and disease leading to growing scientific interest in modulating its composition and subsequent host impacts (Delzenne et al., 2019; Makki et al., 2018). The microbiome is susceptible to alterations caused by aging, changes in the gastrointestinal tract due to shifts in dietary patterns and declines in cognitive function (Hoseini Tavassol et al., 2023). Alterations normally lead to a reduction of bacterial diversity, shifts in dominant species, and overall reduction of beneficial bacteria (Salazar et al., 2017). Contrastingly, dietary interventions like TRF can help restore microbe populations and can help align cellular and molecular pathways throughout the lifespan (Zeb, Wu, Chen, Fatima, Ijaz Ul, et al., 2020).

Factors such as diet composition and genetic makeup are common contributors to obesity (Lin & Li, 2021; Livelo et al., 2023; Noh, 2018). The skeletal muscle (referred to as muscle going forward) accounts for nearly 40%–50% of the normal body mass of humans and accounts for up to 95% of glucose metabolism, making the muscle the largest glucose‐metabolizing organ (Jensen et al., 2011). Under healthy conditions, the muscle regulates insulin‐induced whole‐body glucose disposal while in obesity, compromised muscle physiology can occur leading to insulin resistance and reduced energy levels (Allison et al., 1996; Tran et al., 2019). Further, in obesity, excess fat known as intramyocellular lipids or intramyocellular triglycerides (IMCL/IMTG) can be deposited in the muscle, leading to insulin resistance and tissue dysfunction (Goodpaster et al., 2001; Li et al., 2015). Additionally, muscle in obesity can display reduced mobility, strength, and dynamic balance (Tomlinson et al., 2016). The living microbiome depends on residual food to maintain its community for example *Prevotella* is related to diets rich in carbohydrates and *Bacteroides* may increase in diets in fat and protein (Heintz‐Buschart & Wilmes, 2018).

We have developed *Drosophila melanogaster* (fruit fly) obese flies using a high‐fat diet model (HFD) and a genetically induced model, which lack a functional *sphingosine kinase 2* (*Sk2* mutant) known to lead to accumulation of ceramide and subsequent lipotoxicity (Livelo et al., 2023; Villanueva et al., 2019). Both obese models were also used in uncovering molecular underpinnings of TRF‐mediated benefits in skeletal muscle (Livelo et al., 2023). *Drosophila* models are amenable to studying human metabolic diseases due to their conserved mechanisms associated with nutrient sensing, energy utilization, and energy storage (Chatterjee & Perrimon, 2021). The *Drosophila melanogaster* model serves as an important experimental model for elucidating complex host‐microbiome interactions and has been used by countless studies to model the impacts of microbiota and disease (Erkosar et al., 2013; Ma et al., 2015; Ryu et al., 2010; Storelli et al., 2011). When comparing the digestive system of Drosophila to that of humans, it's notable that Drosophila's arrangement is relatively conserved. It can be segmented into the foregut, midgut, and hindgut, like humans (Chiang et al., 2022).

Studies in both mice and humans support the existence of a gut microbiota‐muscle axis, where human soldiers' stool samples under stress demonstrated an abundance of harmful bacteria with a reduction in anti‐inflammatory microbes while exercised mice altered bacterial composition. (Karl et al., 2017; Przewlocka et al., 2020). The gut microbiota is known for its role in mediating nutrient absorption and aiding in energetic homeostasis (Przewlocka et al., 2020) and can influence muscle function and quality (Shreiner et al., 2015). Due to the gut microbe community's potential role in mediating muscle health in humans and mice, this community may be a target for repairing obesity‐associated muscle dysfunction. Indeed, in humans, bacteria produce several metabolites, which in turn can interact with enteroendocrine cells leading to the creation and release of hormones, which can affect host metabolism (Gribble & Reimann, 2019). These hormones include cholecystokinin, peptide tyrosine, glucagon‐like peptide‐1, glucose‐dependent insulinotropic polypeptide, and 5‐hydroxytryptamine which play a role in pathways involved in insulin sensitivity, glucose tolerance, fat storage, and appetite (Martin et al., 2019). Metabolites abundantly produced by bacteria are the short‐chain fatty‐acids acetate, propionate and butyrate (Topping & Clifton, 2001).

While the interplay between obesity, the microbiome and muscle metabolism has been reported, recent data suggests that TRF is another determinant of microbial community composition in humans leading to substantial changes in the proportion and abundance of microbiota (Zeb, Wu, Chen, Fatima, Haq, et al., 2020). Alterations observed in composition of gut microbe communities that are caused by disorders, such as diabetes, can be reversed by feeding interventions (Karlsson et al., 2013; Zhang et al., 2012). A study evaluating the effects of TRF on microbiota and subsequent influence on nutrient intake was conducted by a recent study in healthy men aged 18‐30 (Zeb, Wu, Chen, Fatima, Ijaz Ul, et al., 2020). The study revealed a robust difference between microbial abundances in genus, phylum, and family levels comparing TRF to non‐TRF groups potentially leading to improvements in metabolism (Zeb, Wu, Chen, Fatima, Ijaz Ul, et al., 2020). Firmicutes and Bacteroides were found to be highly abundant in the TRF group and heat map association revealed that whereas iodine, vitamin E, magnesium, and carbohydrate intake were negatively connected with microbial richness, polyunsaturated fatty acids, and vitamin D were positively correlated with Firmicutes.

Recently, TRF has garnered the attention of the scientific and public community due to its potential as a therapeutic alternative to attenuating the effects of metabolic challenges (Gabel & Varady, 2022; Villanueva et al., 2019; Zhu et al., 2020). The concept behind TRF is a general consolidation of the feeding window where food is consumed only within 6–12 h of the active phase of the day (Charlot et al., 2021; Regmi & Heilbronn, 2020). Sustaining daily rhythms of feeding‐fasting without changes in caloric intake can help promote daily rhythms of gene expression and modulate the composition of gut microbiota (Hatori et al., 2012; Manoogian & Panda, 2017; Zeb, Wu, Chen, Fatima, Ijaz Ul, et al., 2020). Previously, we have shown that TRF attenuated the effects of obesity demonstrated by having improved muscle performance, reduced intramuscular fat, lower phospho‐AKT levels, and displaying a reduced level of an insulin resistance marker (Villanueva et al., 2019). It was uncovered that under genetic‐induced obesity, TRF modulated AMP‐activated protein kinase (AMPK) signaling while in a high‐fat diet, the purine cycle was modulated (Livelo et al., 2023). Further, a human study of 11 obese men in a randomized cross‐over study also showed that imposing a short‐term TRF was sufficient to induce the rhythmicity of lipid metabolism, and amino acids lead to improvements in nocturnal glucose and insulin levels in skeletal muscle (Lundell et al., 2020; Parr et al., 2020). Overall, studies support the beneficial properties of TRF in skeletal muscle, however, the contribution of TRF's modulation of the microbiota and its downstream impact on skeletal muscle physiology remains to be investigated.

Here in this study, we evaluated the influence of removing the gut microbiota on muscle physiology in wildtype *Canton‐S* (CS) and in obese Drosophila models (induced by a HFD or predisposed obesity mutant Sk2) by measuring muscle performance, ATP production, insulin resistance, glucose levels, and ectopic lipid deposition. We also evaluated changes in microbiota composition under TRF and its subsequent impact on the same muscle parameters. We found that removal of microbiota in CS flies (axenic) led to a reduction in overall muscle performance, increased glucose levels, a marker of insulin resistance (*Neural Lazarillo* (Pasco & Leopold, 2012); *NLaz*) and reduced ATP levels. Interestingly, in both obese models, the absence of microbiota improved muscle performance, lowered glucose levels, reduced insulin resistance, and increased ATP levels. Further, axenic CS flies demonstrated increased levels of ectopic lipid density while axenic HFD flies had reduced lipid density and moderate levels of reduction in both lipid density and size in axenic *Sk2*. Using 16S microbiome analysis, we found that the abundance of two bacterial species were consistently modulated by TRF in obese models namely, *Acetobacter pasteurianus* (AP) and *Staphyloccocus aureus* (SA). Both AP and SA interestingly, have been implicated by other studies to have connection through anti‐obesogenic properties (AP) and correlation with increased obesity (SA). We found that TRF led to AP having increased abundance in both obese models while under TRF, SA exhibited reduced abundance in both obese models. Due to previous literature surrounding AP (Fushimi et al., 2006; Kondo et al., 2001; Maruta et al., 2022) and SA Befus et al., 2015) having possible connections with obesity and metabolism impacts on muscle physiology of these two strains were further evaluated. Interestingly, supplementation of AP led to improvements in muscle performance and reduction of glucose levels in CS flies. In contrast, supplementation of SA led to compromised muscle performance and increased overall glucose levels in HFD. Overall, this study provides a novel and unique approach to assessing the effects of microbiota changes on skeletal muscle physiology and highlights TRF's influence on host muscle physiology through the microbiome.

## 2 RESULTS

### 2.1 Removal of microbiota in wildtype led to reduced muscle performance and improvement in muscle performance in obese models

**FIGURE 1:**
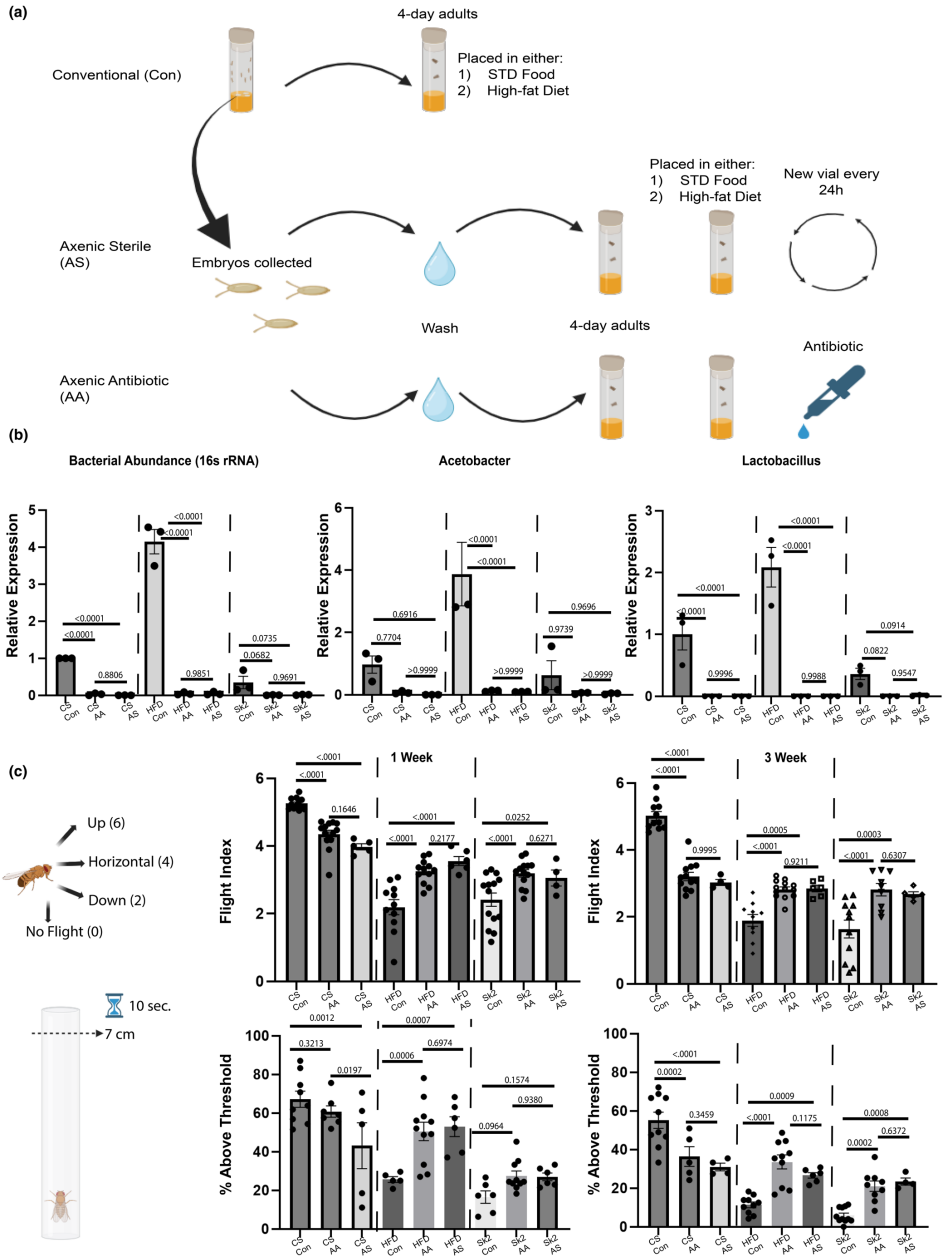
(a) Overall schematic of generating conventional, axenic antibiotic, and axenic sterile flies. (b) Gene expression of 16 s rRNA and two dominant genera (*Acetobacter* and *Lactobacillus*) found in *Drosophila* in all feeding conditions under conventional and axenic status. Both axenic models (antibiotic/sterile) displayed significantly reduced expression compared with conventional control in all three fly models. (c) Image of *Drosophila* flight and climbing assay shown in left. Muscle performance assays (flight and geotaxis) in all feeding conditions and conventional/axenic flies demonstrated reduced performance in CS and improvement in obesity models (HFD/*Sk2*) in females. Results from axenic antibiotic and axenic sterile did not show differences suggesting that differences in muscle performance are due to changes in microbiome. One‐way ANOVA with multiple comparisons done with Uncorrected Fisher's LSD test was performed for both qPCR and muscle assays. *p*‐values are listed with an underline for each bar graph. Each dot represents a cohort of 10–20 flies for the muscle assays. Check source data for details regarding *N* number and flies per cohort and *p*‐values.

We employed two methods of generating microbiota‐free, commonly referred to as axenic flies (antibiotic and sterile based) in standard and high‐fat diet conditions (Figure 1a). Alongside a conventional (microbe community intact) model, two models of axenic flies were generated, one with an antibiotic cocktail and another using frequent transfer of new autoclaved food absent of antibiotic. Two axenic models were initially tested to eliminate the possibility of artifactual effects due to antibiotic supplementation affecting muscle physiology rather than changes in microbiota. Both methods of generating axenic flies were effective in removing bacterial load and did not display any differences in overall effects on muscle performance between both axenic methods (Figure 1b,c). Primer sets for 16S rRNA, Acetobacter, and Lactobacillus (the two most abundant genus found in *Drosophila*) were used to determine the success of axenic models. Two muscle performance assays were employed measuring both the flight index or oxidative muscle capability and climbing ability or glycolytic muscle capability in flies. Overall, CS flies demonstrated reduction in both flight and geotaxis ability in female flies (Figure 1c) and showed similar trends in males (Figure S1a,b). Furthermore, there were no significant differences in muscle performance between axenic status through sterile and antibiotic conditions eliminating the possibility of antibiotic‐induced effects on muscle performance (Figure 1c and Figure S1a,b). Further, studies have shown that leaky gut epithelial layers can lead to inflammation and also be a root cause of alterations in muscle physiology (Nishimura et al., 2020) therefore we employed a SMURF assay using 5‐week‐old flies (Martins et al., 2018), which allows the assessment of leaky gut epithelium presence however, there were no signs of changes between axenic and conventional flies (Figure S1c).

### 2.2 TRF and removal of microbiota in obese models attenuated obesity‐associated deterioration of muscle physiology

**FIGURE 2:**
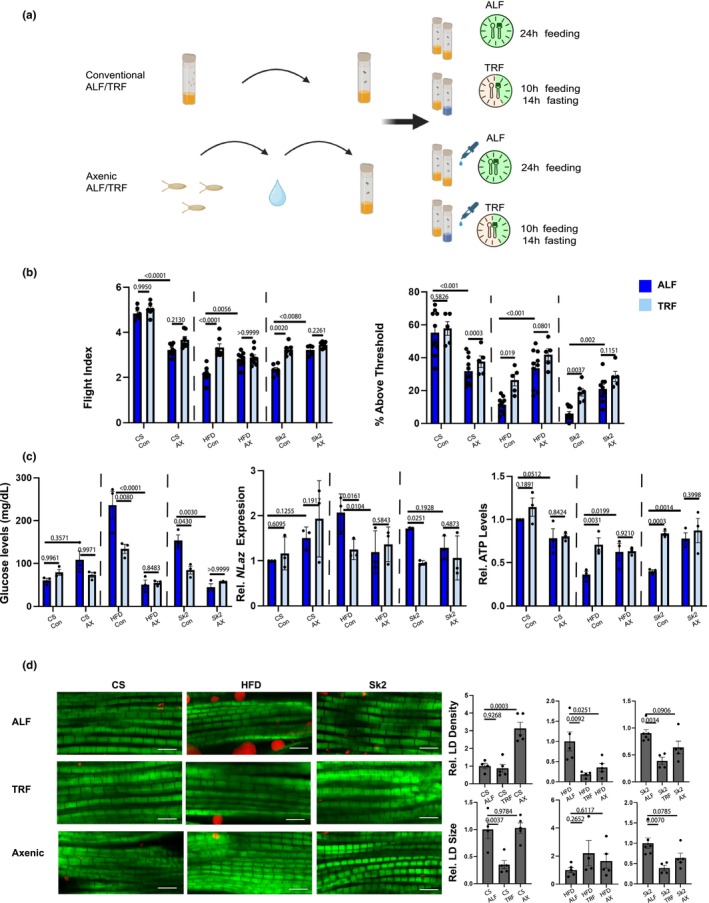
(a) Overall schematic of *Drosophila* grown under ALF and TRF. Conventional ALF flies were allowed 24‐h food access while TRF flies were allowed to eat only the first ZT0‐10 and were transferred to vials with 1.1% agar for the remainder. Axenic flies were also subjected to ALF and TRF with identical feeding/fasting times with the addition of antibiotics in agar for TRF. (b) Muscle performance in 3‐week‐old female flies were assessed. Comparing obese models conventional ALF to axenic ALF also displayed improved muscle performance while CS showed contrasting results. (c) Glucose levels, *NLaz* (insulin‐resistant marker), and ATP levels were measured. TRF in conventional obese models displayed lower glucose levels, lower *NLaz* and increased ATP levels. No changes were observed comparing obese axenic ALF to axenic TRF but comparing conventional obese ALF to obese axenic ALF demonstrated similar trends of improvement as TRF in conventional obese models in metabolic parameters. CS flies contrastingly displayed increased glucose levels, increased *NLaz* and lower levels of ATP but did not show changes in comparing CS axenic ALF to CS axenic TRF. (d) Thoraces were stained with Nile red (lipid) and Phalloidin (f‐Actin). Lipid droplet (LD) density and size were quantified showing the reduction in obese models under TRF and axenic conditions. CS flies under TRF led to reduced size and axenic condition led to increased LD density. A two‐way ANOVA with Uncorrected Fisher's LSD test was performed for both muscle assays and metabolic parameters. One‐way ANOVA with Uncorrected Fisher's LSD test was performed for lipid quantification. *p*‐values are listed with an underline for each bar graph. Each dot represents a cohort of 10–20 flies for (b, c). Data represented from four flies in (d). Check source data for my information regarding the total N number and *p*‐values.

Flies were raised under both ALF and TRF in either conventional or axenic conditions in all three fly models (CS, HFD, and *Sk2*) (Figure 2a). Conventional flies were similarly raised on the standard diet until day 4 after which flies were either assigned to ad libitum *feeding* (ALF) or TRF conditions. ALF flies had 24‐h food access and TRF only had 10 h. food access followed by 14 h of fasting. Additionally, flies raised in axenic conditions involved embryo collection, washing, and the final addition of antibiotics in both ALF and TRF conditions. We found improvements under TRF similar to previous studies (Villanueva et al., 2019) in both HFD and *Sk2* in 3‐week‐old females in flight and geotaxis performance (Figure 2b). There were no notable differences in comparing ALF axenic flies to TRF axenic flies apart from CS flies suggesting that in CS flies, microbiome is necessary for noticeable improvement (Figure 2b). Interestingly, in obese conditions TRF led to improved muscle performance in axenic TRF conditions and ALF axenic conditions suggesting pleiotropic benefits microbial effects (Figure 2b). We found that measurement of glucose levels, *Neural Lazarillo* (*NLaz*; an insulin resistance marker) (Pasco & Leopold, 2012) and ATP levels did not show significant differences in TRF compared to ALF under axenic conditions in all models (Figure 2c). Trends found previously in muscle performance (Figure 1b) showed similar improvements in metabolic parameters in comparing axenic ALF obese flies to conventional ALF obese flies. We observed lower glucose levels and *NLaz* expression in addition to higher ATP levels in axenic ALF obese flies compared to conventional ALF obese flies (Figure 2c). Lipid deposition was also evaluated using Nile‐Red, we observed reductions in either or both lipid droplet (LD) density or size in conventional TRF flies compared to conventional ALF flies (Figure 2d). Interestingly, we also found similar improvements comparing ALF axenic flies in obesity compared to ALF conventional counterparts (Figure 2d). CS in TRF demonstrated lower lipid size droplets compared to ALF conventional flies (Figure 2d). While ALF CS in the axenic condition displayed increased LD density compared to ALF conventional CS (Figure 2d). HFD showed drastic reductions in lipid density in both TRF, and axenic flies compared to ALF conventional flies while *Sk2* showed reductions in both density and size in TRF and axenic models. Additionally, we measured canonical immune‐associated gene expression in *Drosophila* (d*Stat5A*, d*TNFa*, *Imd*) (Figure S2a) to determine the potential contributions of inflammation to the observed phenotypes. However, no trends were observed in ALF and TRF under axenic and conventional conditions in all 3 models. We also examined trends in TRF using previous sequencing data in fly muscle (Villanueva et al., 2019), which suggest possible changes in glucose regulatory pathways (Figure S2b). We found a modest decrease in gene *Glucose‐Transporter 1* (*Glut1*) under TRF in HFD and a reduction in energetic regulator *PPARG coactivator 1 alpha* (*Ppargc1A*) in *Sk2* and increases in *Acetyl‐CoA carboxylase 2* (*Acc2*) a gene involved in fatty synthesis and oxidation (Figure S2b). We found little change in *Myogenic differentiation 1* (*Myod1*) involved in myogenic differentiation and *Forkhead box protein O1* (*FoxO1*) only in *Sk2* under TRF (Figure S2b). Although we did not have data on axenic models, this dataset may suggest that TRF related changes in microbiota led to modulation of these proposed key genes. Additional studies needed to establish possible associations of the microbiome and TRF to changes in these expressions (Figure S2b).

### 2.3 TRF led to an increased abundance of *A. pasteurianus* and a reduced abundance of *S*. *aureus* in obese models

**FIGURE 3:**
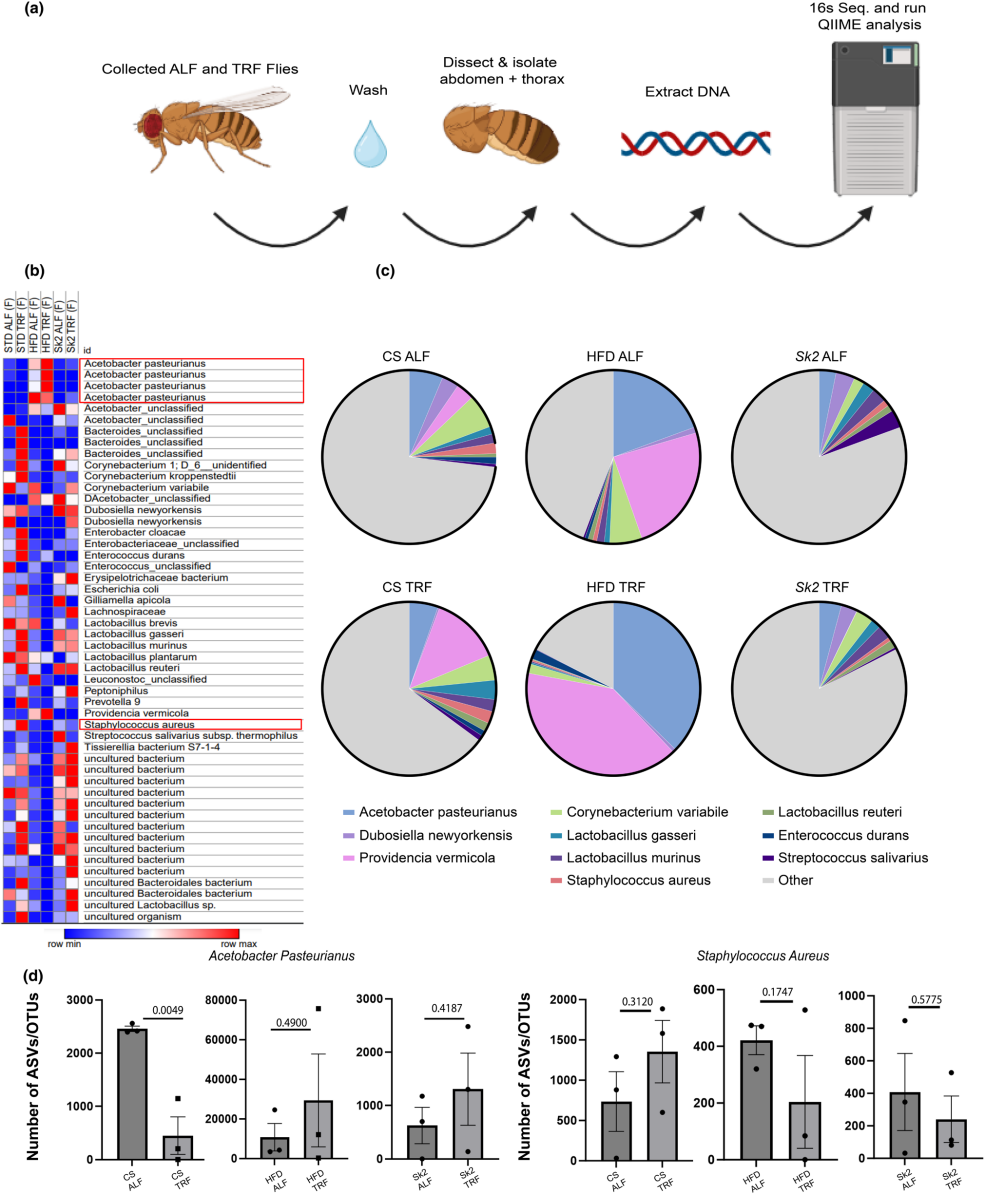
(a) Overall schematic depicting ALF and TRF flies going through a wash step (70% EtOH and water) beforethe isolation of midgut from female and male fly abdomen and thorax and extraction of DNA. Analyses were run by the UAB microbiome core using “Quantitative Insights into Microbial Ecology” (QIIME) analysis (b) A heatmap displaying the relative abundance of bacterial species in ALF/TRF conditions in female flies. *Acetobacter Pasteurianus* (AP) showed an increase in both obese models and *Staphyloccocus aureus* (SA) showed a decrease of abundance under TRF which were further evaluated in female flies. (c) Pie charts showing the top bacterial abundances found in all 3 fly models under ALF and TRF in female flies. (d) Bar graphs of amplicon sequence variants in females (ASVs) of AP show a general trend of increasing abundance while SA demonstrated a general reduction under TRF. *p*‐values were obtained using a student unpaired *t*‐test. Check source data for my information regarding the total *N* number and *p*‐values.

To compare differences in microbiome between ALF and TRF, both TRF and ALF fly midguts were isolated by collecting abdomen and thorax from both male and female flies at 3 weeks of age (Figure 3a). A heatmap plotting the relative abundances from top amplicon sequence variant (ASV) reads are shown (Figure 3b) representing the top bacterial species. Although other species showed distinct modulation under TRF, we found two bacterial species that had consistent increase/decrease under both obese models. These two species included *A. pasteurianus* (AP) increasing in obese models and *S. aureus* (SA) decreasing in obese models under TRF indicated with a red box (Figure 3b). Literature regarding AP has been demonstrated to have possible properties of anti‐hyperglycemic (Salbe et al., 2009), anti‐hypertensive (Kondo et al., 2001), anti‐cholesterol (Fushimi et al., 2006) and anti‐obesity (Kondo et al., 2009) as an acetic acid producing bacteria. Furthermore, AP had one of the highest abundances in the top 10 bacterial species in our 3 models (Figure 3c). Interestingly, SA is associated with having increased colonization amongst obese humans (Befus et al., 2015) yet not much has been explored regarding its downstream impacts on metabolism and obesity. SA was also a part of the top 10 relative abundance of species from the three models and was notably reduced in both obese models under TRF (Figure 3c). TRF increases in AP in obese models can be seen from the ASV count in addition to reduction in SA in obese models (Figure 3d). Both AP and SA however, did not display the same pattern in males (Figure S3a) but still displayed high abundance in ALF and TRF (Figure S3b). Overall, TRF trends suggest that AP and SA may have female specific effects and further investigation will be needed to explore key bacteria in male flies which modulate muscle physiology.

### 2.4 Supplementation of *A. pasteurianus* and *S. aureus* improve and deteriorate muscle physiology respectively

**FIGURE 4:**
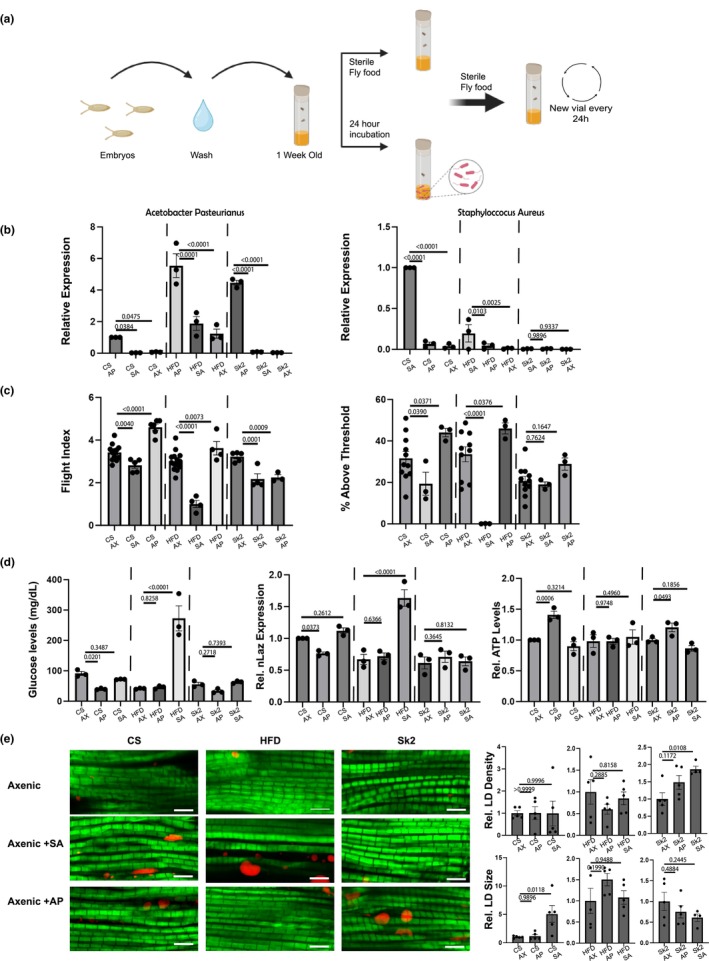
(a) Overall schematic depicts axenic sterile flies at 1 week of age either being supplemented with bacteria for 24 h in standard or HFD food (light brown vs. dark brown respectively) or just being maintained in sterile food as a control in standard/HFD food. After the 24‐h incubation period, both groups were maintained on sterile food and changed every 24‐h. (b) qPCR was performed to measure the relative expression of flies either supplemented with AP/SA or flies maintained on sterile axenic food. The primes used for measuring AP showed CS supplemented with AP with the highest expression compared to others and similarly in HFD and *Sk2* supplemented with AP. SA also demonstrated higher expressions in CS and HFD supplemented with SA. (c) Muscle performance assays displayed improvement in CS and HFD flies which were supplemented with AP. CS, HFD, and *Sk2* displayed a reduction in muscle performance when supplemented with SA. (d) CS flies supplemented with AP led to lower glucose levels, lower *NLaz* expression and higher ATP levels in both CS and *Sk2*. HFD flies supplemented with SA led to increased glucose levels and *NLaz* expression. (e) Indirect flight muscle was stained with nile red (lipid) and Phalloidin (f‐Actin). Lipid droplet (LD) density and size were quantified in flies either in axenic sterile condition or flies supplemented with either AP or SA. Only CS and *Sk2* flies supplemented with SA displayed changes in lipid quantification shown by increased LD size in CS and increased density in *Sk2*. One‐way ANOVA with Uncorrected Fisher's LSD test was performed for qPCR, muscle assays, metabolic parameters and lipid quantification. *p*‐values are listed with an underline for each bar graph. Each dot represents a cohort of 10–20 flies for (b–d). 1 Dot represents one fly in bar graphs in (e). Check source data for my information regarding the total *N* number and flies per cohort in addition to *p*‐values. Furthermore, additional comparisons were made including conventional counterparts of WT, HFD and *Sk2* shown in source data.

To test the impact of both bacterial species, axenic sterile flies either supplemented with AP or SA and compared with non‐supplemented sterile axenic flies (Figure 4a). The non‐supplemented group was maintained in sterile conditions and changed with newly autoclaved food every 24 h while the supplemented group had either AP or SA added to the food for 24 h. After 24‐h supplementation, both groups were maintained on sterile autoclaved food and changed every 24 h similar to the non‐supplemented group (Figure 4a). We performed qPCR measurement of flies post‐supplementation to verify the success of the supplementation. From the results (Figure 4b), AP was successful for all three models; however, SA did not show high abundance after supplementation in *Sk2* (Figure 4b). From transcriptomic data in *Drosophila* muscle, we found a group of lysozyme genes (a strong modulator of bacterial populations in *Drosophila* (Marra et al., 2021) to be increased in *Sk2* comparatively to other models (Figure S4a).

Interestingly, supplementation with AP improved muscle performance in the CS and HFD models, as evidenced by enhanced flight and geotaxis abilities. In contrast, flight performance decreased in the Sk2 model, with minimal changes observed in geotaxis ability (Figure 4c). On the other hand, supplementation with SA reduced flight performance across all three models, while only CS and HFD showed reduced geotaxis ability (Figure 4c). In the CS model, AP supplementation also lowered glucose levels and decreased NLaz expression (Figure 4d). However, AP did not affect glucose levels in the HFD and Sk2 models (Figure 4d). Additionally, AP supplementation increased ATP levels in both CS and Sk2 models (Figure 4d). The effects of AP and SA supplementation on ectopic lipid deposition were also assessed. Overall, AP supplementation did not alter lipid density or size in any of the three models. In contrast, SA supplementation resulted in increased lipid droplet (LD) size in the CS model and increased LD density in the Sk2 model.

## 3 DISCUSSION

The changes in the microbiome associated with TRF reveal untapped potential and provide an opportunity to deepen our understanding of the mechanisms behind TRF's benefits. Metabolic challenges including obesity continue to be an ongoing issue globally, which has led to hampering healthcare and major economic burdens (Reges et al., 2020). As the muscle is the largest metabolic organ, the knowledge behind preserving muscle physiology is essential to developing therapeutic programs that seek to restore muscle physiology and attenuate the pathophysiological impacts of prevalent metabolic challenges (Guo et al., 2021; Livelo et al., 2023; Mensink et al., 2007; Villanueva et al., 2019). The majority of the muscle's physiological role is tied to its contributions to energy production and metabolism including glucose uptake and storage in addition to essential tasks such as breathing and movement (Klumpp et al., 2010). Skeletal muscle physiology is influenced by various factors, including diet, genetics, meal timing, and changes in gut microbiota populations (Campion et al., 1984; Gizard et al., 2020; Poggiogalle et al., 2021; Ticinesi et al., 2017; Villanueva et al., 2019). While TRF has been demonstrated to modulate gene expressions of metabolic pathways in the muscle in HFD and *Sk2* (Livelo et al., 2023), TRF effects on microbiota and subsequent impacts on muscle physiology demonstrate a need to be further explored.

**FIGURE 5:**
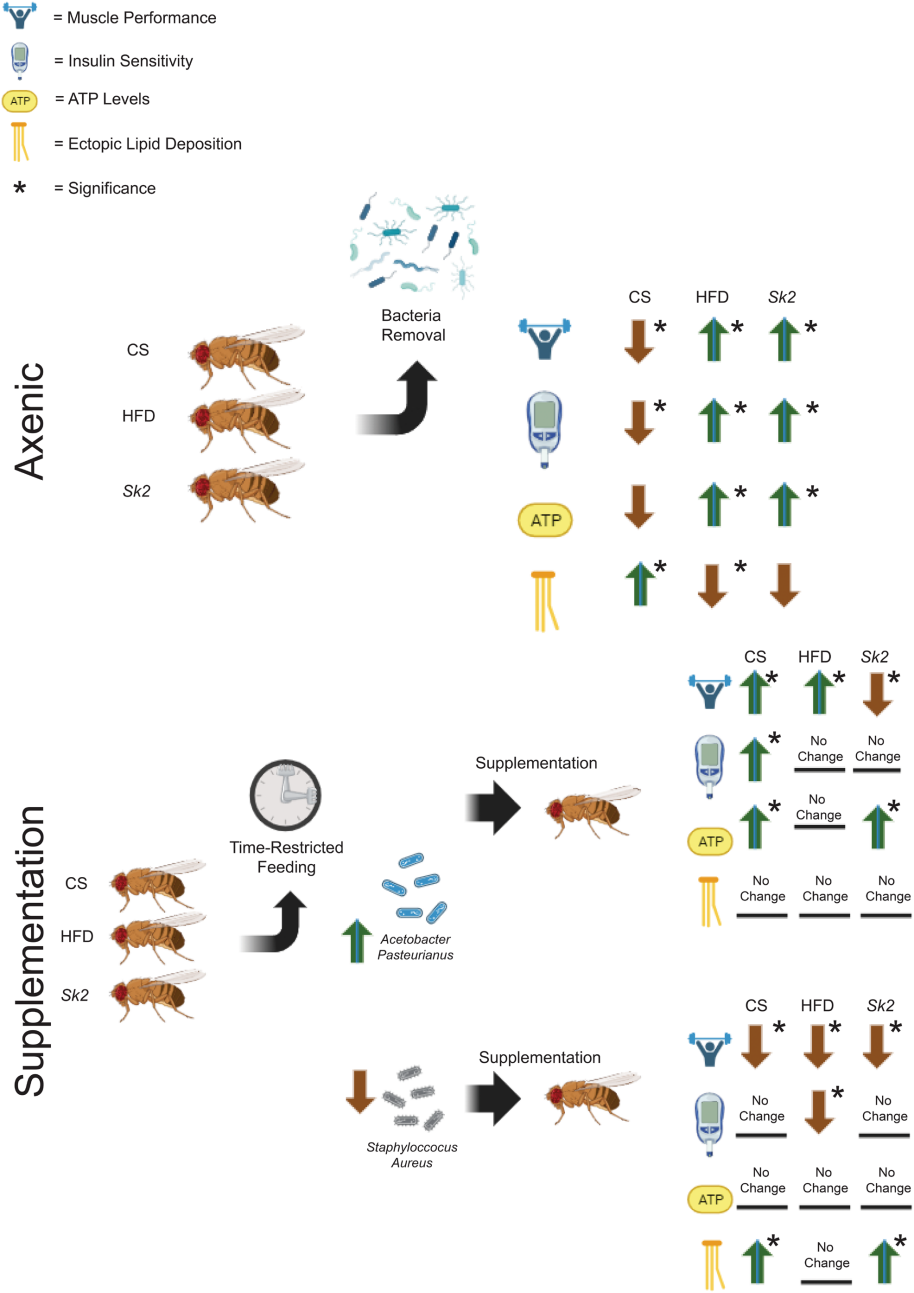
Graphical illustration showed the impacts of microbial removal and supplementation of TRF‐associated bacteria in muscle performance and metabolic parameters. The top panel result shows that upon removal of microbiota (Axenic) in wild‐type (CS) or obesity models, (HFD, and *Sk2*) have opposite effects on muscle performance and alteration of other metabolic parameters (up and down arrows). The bottom panel shows the impact of each microbiome supplementation and TRF on muscle performance and other metabolic parameters in wild‐type or obesity models (up and down arrows). Introduction of TRF in wild‐type or obesity models led to increase in *Acetobacter pasteurianus and* decrease *Staphyloccocus aureus*. Subsequently, supplementing flies with either *A. pasteurianus* or *S. aureus* led to significant alteration in muscle performance and other metabolic parameters.

Interestingly, utilizing a supplementation assay to explore effects on muscle physiology from TRF led to key insights. Adding AP led to improved muscle performance in CS and HFD while reducing total glucose levels, insulin resistance marker in CS and increasing ATP in CS and *Sk2* (Figure 4c,d). Meanwhile, Supplementing SA, led to reduced muscle performance in all three models and increased glucose levels and insulin resistance markers in HFD (Figure 4c,d). The addition of SA also led to increased lipid deposition in *Sk2* and CS (Figure 4e). A graphical conclusion representing all highlights of this study can be found in Figure 5a,b. It is noteworthy that after multiple attempts, we were not able to increase SA abundance in *Sk2* through supplementation, which may suggest a defense response against exogenous addition of a pathogenic strain specifically in the *Sk2* model. Although not conclusive, this may suggest that this increased lysosomal response may prevent the proliferation of a pathogenic bacterial species such as SA in *Sk2*. Additionally, the lower abundance of SA in CS compared to HFD suggests that further investigation is needed to understand its specific effects on *Sk2*.

While there is little information regarding TRF and microbiota effects on muscle, it is known that microbiota‐related changes can influence the skeletal muscle through the regulation of various processes involved in inflammatory and immune response, anabolism of protein, energy and lipid metabolism, insulin resistance, and others (Lustgarten, 2016; Przewlocka et al., 2020; Shreiner et al., 2015). Measurement of immune‐related genes in 3‐week female muscle in *Drosophila* did not display any consistent trends between ALF and TRF and conventional and axenic flies (Figure S2a) but changes in other pathways may have led to modulations in muscle physiology. Acetic acid‐producing bacteria (AAB) such as AP have demonstrated in recent studies to confer anti‐obesogenic properties (Kondo et al., 2001; Kondo et al., 2009) in addition to anti‐hyperglycemic properties (Salbe et al., 2009). Interestingly, previously collected muscle transcriptome from 3‐week female flies displayed *Glut1* exhibited a modest reduction in HFD TRF with increases in *Acc2* in both HFD and *Sk2* (Figure S2b). Further, *FoxO1* which may lead to AMPK activation was upregulated in *Sk2* with increased *AMPK* expression (Figure S2b). This result was also like a study in male Sprague Dawley rats, which demonstrated that supplementation of acetic acid, activated AMPK and prevented a decrease in type 1 muscle fiber loss (Maruta et al., 2022). Although these results were shown in flies under TRF, further studies measuring expressions of metabolic genes in flies with bacterial supplements are needed to draw further conclusions regarding the associated changes.

Overall, this study provides a novel approach to studying the impacts of the removal of the natural microbiota and the supplementation of specific bacterial species to study their contributions to muscle physiology. This study demonstrated that removing the gut microbiota in wildtype led to deterioration in muscle physiology while removal of microbiota under obese conditions intriguingly led to improvements in muscle performance and some parameters of metabolism. Further, while employing TRF, a known behavioral intervention historically attenuated the effects of obesity on muscle, this study focuses on TRF‐mediated changes to the microbiome and its host physiology. Results showed changes in bacterial species, notably AP and SA. Supplementation of AP led to benefits in muscle performance in CS and HFD while also improving metabolic parameters in all three models. Further, SA supplementation led to opposing changes, which reduced muscle performance in all models and led to deterioration of metabolic parameters like ectopic lipid deposition in *Sk2*.

Our study has some limitations that will be addressed in future work. Comparisons of ASVs were primarily conducted without considering alpha and beta diversity parameters. Future experiments with greater statistical power will evaluate these parameters, and the effects on microbial communities can be more thoroughly explored with larger cohort sizes. Although TRF experiments showed clear effects, using a 1.1% agar concentration may not completely rule out the possibility of flies consuming limited nutritional content. Additionally, while a capillary feeding assay (CAFÉ) was previously conducted between ALF and TRF flies with microbiomes, this assay has not yet been tested on axenic flies.

## 4 MATERIALS AND METHODS

### 4.1 Drosophila models, diets, and feeding fasting regimens

As previously described (Villanueva et al., 2019), Canton‐S and Sphingosine kinase 2 (*Sk2* BDSC: 14133) were obtained from Bloomington *Drosophila* Stock Center (BDSC) and maintained at 25°C. *Sk2* is a *Drosophila* ortholog of human *Sphingosine kinase 1. Sk2* loss‐of‐function mutant has been used in previous obesity‐related studies in *Drosophila* and has demonstrated to lead to accumulation of ceramide, implicated to contributing towards obesity (Livelo et al., 2023; Villanueva et al., 2019; Walls et al., 2018). The standard diet used for fly vials includes agar 11 g/L, active dry yeast 30 g/L, yellow cornmeal 55 g/L, molasses 72 mL/L, 10% nipagen 8 mL/L and propionic acid 6 mL/L. A temperature of 121 degrees Celsius was used for autoclaving the food. HFD model used the same components as the standard diet with the addition of 5% coconut oil as previously established (Villanueva et al., 2019). CS and *Sk2* flies were only fed with the standard diet while CS fed with 5% coconut oil were used for HFD. To maintain axenic conditions, antibiotics were added to the standard diet, which includes a cocktail of four antibiotics (Ampicillin/Kanamycin/Tetracyclin/Erythromycin at 50 mg/mL final each) and for axenic sterile conditions standard diet was used and changed every 24‐h instead of every 5 days in all other conditions.

### 4.2 Bacterial strains and quantification

The *A. pasteurianus* (AP) strain was received from Dr. Edan Foley's lab and was grown using an MRS plate for 2–3 days at 29°C and MRS broth for 2 days static 29°C. Supplementation of AP was done at 4.0 × 10^7^ CFU by adding 50 μL of culture on the food surface of sterile fly food vials for 24 h. *S. aureus* (SA) #29213 was purchased from American Type Culture Collection (ATCC) and was grown on tryptic soy broth/plates. SA was grown at 37°C static for 24 h. SA was supplemented at 5.0 × 10^7^ CFU by adding 50 uL of culture on the food surface of fly food vials for 24 h. The success of axenic models was determined by measuring the expression of 16 s rRNA and Acetobacter and Lactobacillus genus primers. Before the isolation of midguts, flies were briefly washed in 70% EtOH and briefly rinsed with milliQ water. 10 Midguts were isolated from 3‐week‐old female flies and DNA was extracted with DNEasy Ultraclean Microbial kit from Qiagen (catalog # 12224–50) and 5 ng of pure DNA was used per qPCR reaction. All primers used for qPCR are included in the source table.

### 4.3 Muscle performance

#### 4.3.1 Flight index (oxidative)

To assess the contributions of TRF and microbiome to muscle performance, a flight index assay was carried out as previously described (Drummond et al., 1991; Villanueva et al., 2019). In brief, this methodology involves releasing both control and experimental adult flies into the center of a Plexiglas chamber with an overhead light source. Flies were introduced in groups of 10–20 individuals. Flight indices (FI) were assigned based on each fly's capability to ascend [6.0], move horizontally [4.0], descend [2.0], or not fly at all [0.0]. 1 and 3‐week aged flies were used for this having at least 3 cohorts with at least 10–15 flies per cohort.

### 4.4 Negative geotaxis (glycolytic)

As described in a prior study (Villanueva et al., 2019), flies were moved to a fresh vial (with 10–13 flies per trial using at least 3 biological replicates per condition) and given a 2‐min period to acclimate. Subsequently, the vial underwent three taps to induce a negative geotaxis reaction. The flies' climbing behavior was recorded on video for later analysis. At 10‐s intervals, the proportion of flies that successfully reached the 7 cm mark was recorded (Villanueva et al., 2019). Analysis for both assays.

### 4.5 Myofibril organization and ectopic lipid deposition

As previously outlined (Villanueva et al., 2019) for cytological examination, specimens were fixed in 4% PFA, rinsed with PBS, longitudinally aligned (thoraces) in a cryomold containing Tissue‐Tek OCT (Sakura), and swiftly frozen on dry ice. Sections of 30 μm thickness were obtained through cryosectioning, followed by washing and staining with 1× fluorescence dye 488‐I labeled with Phalloidin (1 μg/mL) to detect structural abnormalities and Nile red (1 μg/mL) in 1× PBS for lipid staining. Fluorescent images were captured using an Olympus BX‐63 microscope with cell‐tracking software. Lipid droplets were quantified by measuring their size and density using Fiji ImageJ (version 1.54f). This process involved applying Otsu thresholding and watershed segmentation to the images.

### 4.6 ATP quantification

ATP levels in the thoraces of 3‐week‐old flies were assessed using a luciferase‐based ATP kit from Thermo Fisher. The ATP collection protocol followed a previously established method (Tennessen et al., 2014). To summarize, fly thoraces were homogenized with a homogenization buffer (consisting of 6 M guanidine HCl, 100 mM Tris at pH 7.8, and 4 mM EDTA) using a pellet pestle. A small portion of the homogenate was utilized for protein level determination via a Bradford assay from Bio‐Rad. The remaining homogenate was subjected to boiling for 5 min, followed by centrifugation at maximum speed for 3 min at 4°C. The luciferin and luciferase were appropriately diluted as per the ATP kit instructions, and ATP‐dependent luminescence was measured at 560 nm. Triplicate measurements were taken for each condition, and an average was computed after three consecutive readings. These values were then normalized based on protein levels. Both protein and ATP levels were determined using the EPOCH Biotek plate reader three biological replicates were assessed using seven flies each.

### 4.7 16S microbiome analysis and bioinformatics

ALF and TRF flies underwent a wash step (70% EtOH and water) before isolation of midgut from the abdomen and thorax and extraction of DNA. The isolated DNA was then used for PCR or stored in Tris‐EDTA buffer for later use. The DNA was quantified in a Spectrophotometer before PCR. PCR amplification of the V4 region of the 16S rRNA gene was carried out using unique barcoded primers to create an amplicon library. The individual PCR products were run on an Agarose gel, bands visualized by UV illumination, and they were in turn excised and purified by QIAquick Gel Extraction Kit (Qiagen, Germantown, MD, USA). The PCR products were sequenced using the Illumina MiSeq platform by 250 bp paired‐end sequencing (Kumar et al., 2014; Van Der Pol et al., 2019).

Demultiplexed data files were obtained from the UAB Microbiome Resource following the sequencing process. The initial stage of analysis involved importing FASTQC files into the Quantitative Insights for Microbial Ecology 2 (QIIME2) environment as qza files using the “import” function and the manifest method. Subsequently, the DADA2 denoising algorithm was applied to cluster sequences at 99% similarity. The DADA2 workflow encompasses several key steps: read filtering, dereplication, chimera removal, and merging of paired‐end reads. The ultimate outputs from DADA2 include an Amplicon Sequence Variant (ASV) table and a file containing representative sequences corresponding to ASV IDs.

### 4.8 Real‐time quantitative PCR

The thoraces or indirect flight muscles (IFMs) of 3‐week‐old flies were isolated and rapidly frozen. RNA extraction was performed using the Zymo Research Quick‐RNA Microprep Kit, which included on‐column DNase I digestion. Quantitative PCR was conducted using the SsoAdvanced Universal SYBR Green supermix from Bio‐Rad, employing the BIO‐RAD CFX Opus Real‐Time PCR System. Expression levels were standardized using the 60S ribosomal protein (RPL11) as a reference three biological replicates were used with eight flies each.

### 4.9 Glucose quantification

Briefly, using 10 flies per condition with 3 biological replicates were used for thorax isolation. Glucose was measured using Wako's LabAssay Glucose Kit (Mutarotase‐GOD method) in a 96‐well plate. The plate was incubated for 30 min at 37°C and read immediately at 340 nm. The free glucose concentration was measured by comparing free glucose measurements for each sample to the glucose standard curve.

### 4.10 Statistical analysis

Statistical significance in flight muscle performance and climbing ability was assessed through either one‐way ANOVA or two‐way ANOVA, followed by Fisher's LSD test. Differences in lipid droplet size and density were evaluated using one‐way ANOVA followed by Fisher's LSD test. Metabolic parameters were calculated using two‐way ANOVA, followed by Fisher's LSD test. A student *t*‐test was run to compare ALF and TRF‐related abundances in AP and SA. All tests were performed with GraphPad Prism 10.

## AUTHOR CONTRIBUTIONS

GCM and CL designed the experiments. CL prepared samples for 16S Microbiome Sequencing. CL analyzed 16S Microbiome Sequencing data with help from CM. CL performed all the muscle functions and with JM and YM collected cytological data. CL performed analysis, acquired biochemical and metabolic data, and analyzed all the data including statistical analyses with help from GCM and CM. CL prepared the paper with GCM's input. All authors provided feedback on the revised manuscript.

## FUNDING INFORMATION

This work was supported by National Institutes of Health (NIH) grants AG065992 and RF1NS133378 to G.C.M. This work is also supported by UAB Startup funds 3,123,226 and 3,123,227 to G.C.M.

## CONFLICT OF INTEREST STATEMENT

The authors declare no competing interests.

## Supporting information


**Data S1:** Source data.

Data S2:

## Data Availability

The data supporting this study's findings are available from the corresponding author upon reasonable request.
