# Supplementary material for: Time‐restricted feeding mediated modulation of microbiota leads to changes in muscle physiology in Drosophila obesity models
Source: Aging Cell. 2024 Oct 24;24(2):e14382. doi: 10.1111/acel.14382 (PMC11822661; doi:10.1111/acel.14382)
Supplement: Supplementary file 2 — Data S2: [file ACEL-24-e14382-s002.pdf]

Supplementary Materials for

Time-restricted feeding mediated modulation of microbiota leading to changes in muscle physiology in Drosophila obesity models

Melkani et al., Correspondence and requests for materials should be addressed to G.C.M
(Email: girishmelkani@uabmc.edu)

This PFD file includes:
Supplementary Figures 1 to 4

Supplementary Figure 1

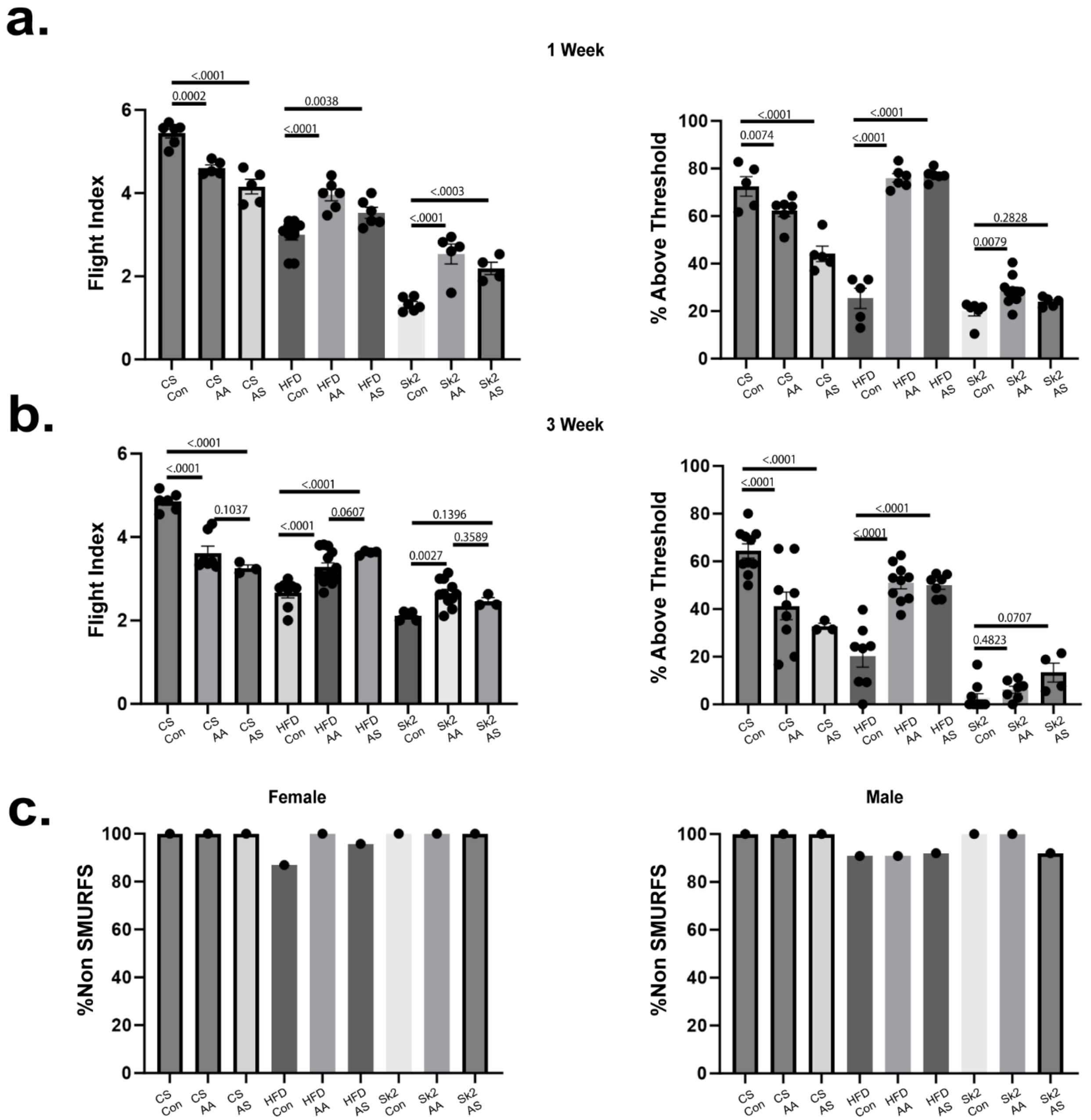

Supplemental Figure 1: **a)** Muscle performance assays (flight and geotaxis) in all feeding conditions and conventional/axenic flies demonstrated reduced performance in CS and improvement in obesity models (HFD/*Sk2*) in 1-week males and **b)** 3-week males. **C)** SMURF assays were performed to test if the integrity of the epithelial layer was compromised, but results did not display differences between models. One-way ANOVA with Fisher's LSD test was performed for muscle assays. P-values are listed with an underline for each bar graph. Each dot represents a cohort of 10-20 flies for muscle assay and one dot represents a cohort of 30-40 flies in SMURF assay.

Supplementary Figure 2

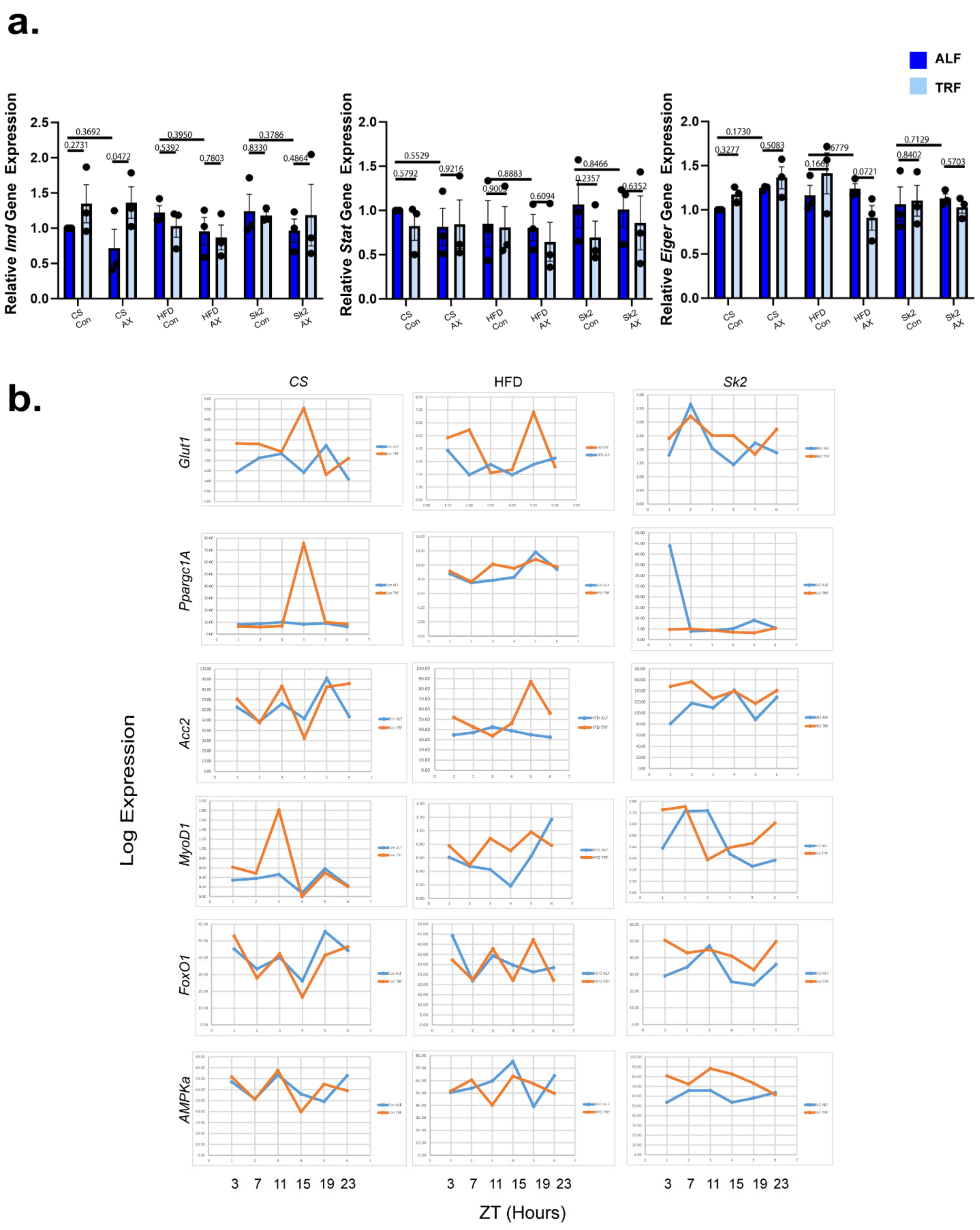

Supplemental Figure 2: a) qPCR was performed to measure the expression of canonical inflammatory genes in *Drosophila.* No significant trends or differences were observed for the inflammatory genes in all conditions including axenic and conventional ALF/TRF flies. **b)** Muscle transcriptomic data was examined for genes involved in metabolic pathways in ALF/TRF. *Glut1* (glucose transporter) demonstrated a moderate reduction in HFD TRF, *Ppargc1A* (energy metabolism) was reduced in *Sk2* TRF, and *Acc2* (fatty oxidation) was increased in HFD TRF with moderate increases in CS and *Sk2* in TRF. *MyoD1* (muscle cell differentiation) led to moderate increases in CS in TRF, *FoxO1* (metabolism, adaptive immunity) increased in *Sk2* in TRF, and *AMPK* (energy metabolism) increased in TRF. Two-way ANOVA with Fisher's LSD test was performed for inflammatory genes, with each dot representing a cohort of 10 flies; the P-value is listed.

Supplementary Figure 3

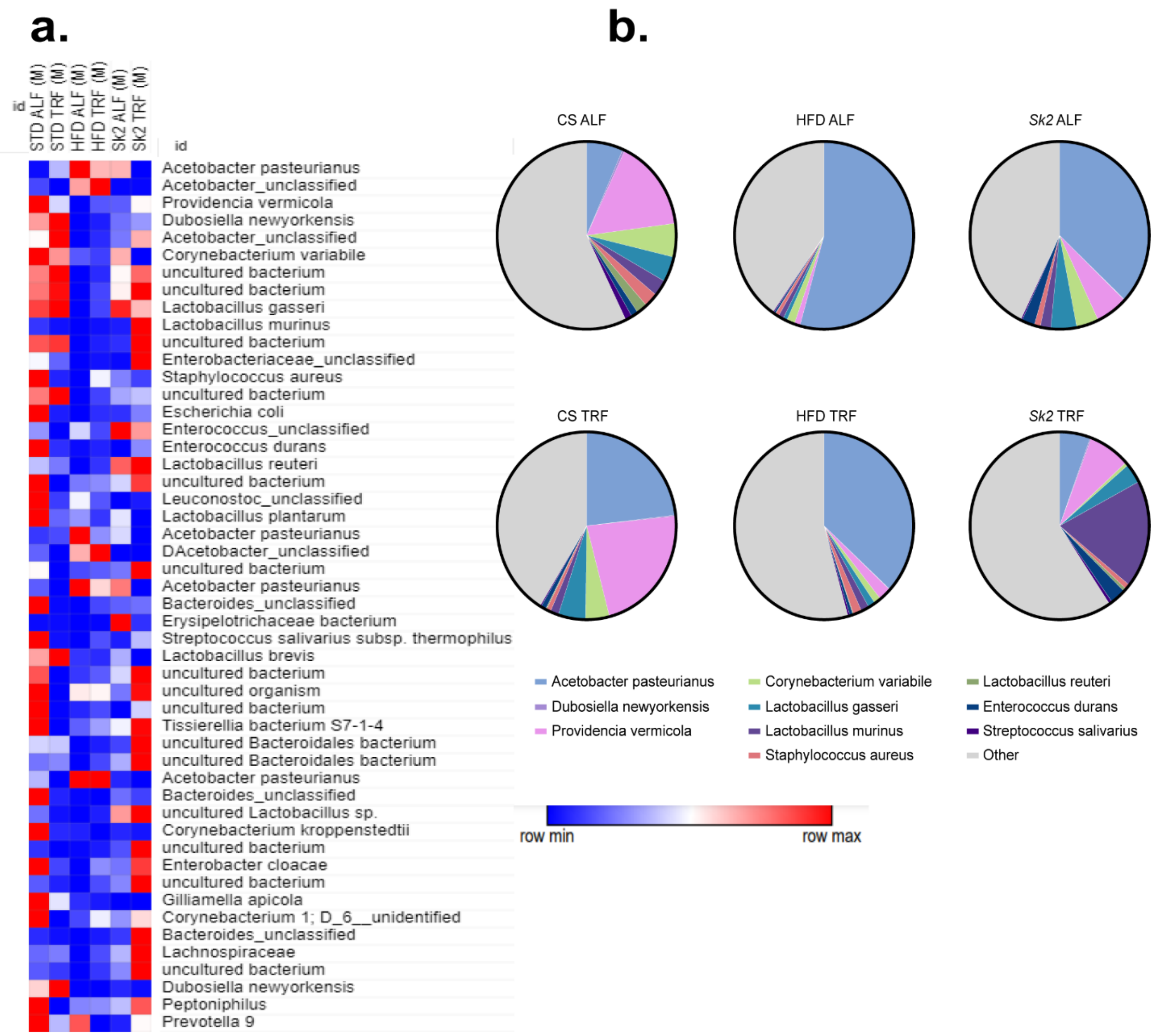

Supplemental Figure 3: a) A heatmap displaying the relative abundance of bacterial species in ALF/TRF conditions. Patters exhibited in females did not exhibit the same trends as seen in 3-week males. **c)** Pie charts showing the top 10 bacterial abundances found in all 3 fly models under ALF and TRF.

Supplementary Figure 4

a.

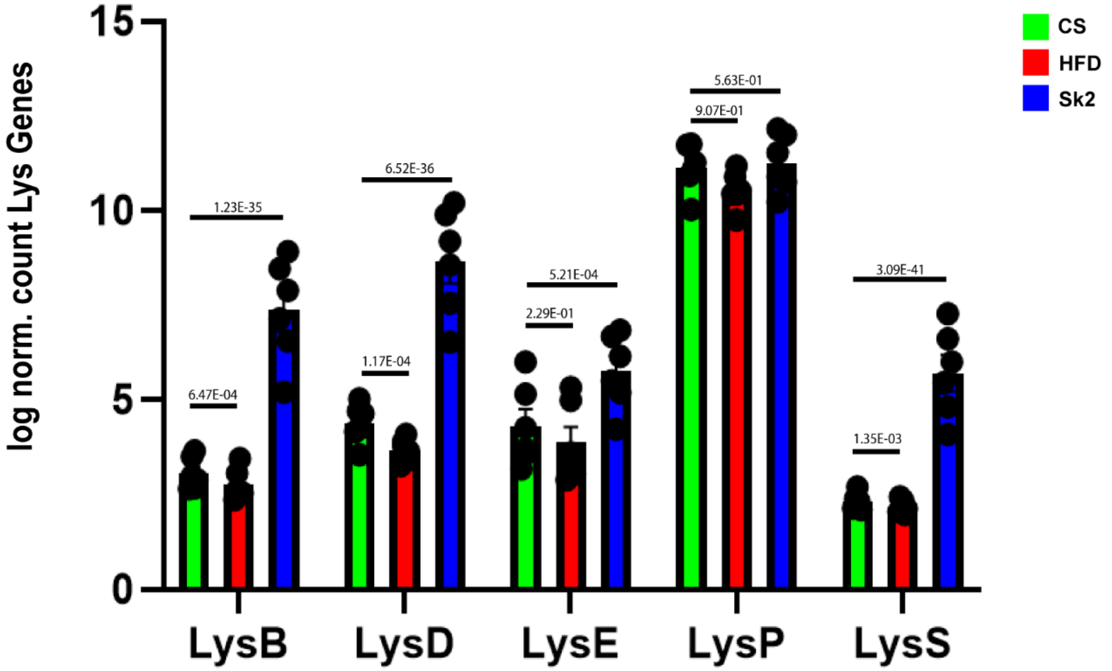

Supplemental Figure 4: a) Expressions of lysosomal genes potentially responsible for regulating bacterial species proliferation from muscle transcriptome data done in 3-week-old female flies. *Sk2* demonstrated higher levels of *Lys B, D, E,* and *S* compared to other models under ALF potentially preventing supplementation of a pathogenic bacteria such as SA. Each dot represents a cohort of 10 flies.
